# Microbial and enzymatic activity of soil contaminated with a mixture of diflufenican + mesosulfuron-methyl + iodosulfuron-methyl-sodium

**DOI:** 10.1007/s11356-014-3395-5

**Published:** 2014-08-07

**Authors:** Małgorzata Baćmaga, Agata Borowik, Jan Kucharski, Monika Tomkiel, Jadwiga Wyszkowska

**Affiliations:** University of Warmia and Mazury in Olsztyn, Plac Łódzki 3, 10-727 Olsztyn, Poland

**Keywords:** Diflufenican, Mesosulfuron-methyl, Iodosulfuron-methyl-sodium, Microorganisms, Biodiversity, Enzymes, Resistance

## Abstract

The aim of this study was to determine the effect of three active substances, diflufenican, mesosulfuron-methyl and iodosulfuron-methyl-sodium, applied in combination, on soil microbial counts, the structure of soil microbial communities, activity of soil enzymes and their resistance to the tested product, the biochemical indicator of soil fertility, and spring wheat yield. Soil samples with the granulometric composition of sandy loam with pH_KCl_ 7.0 were used in a pot experiment. The herbicide was applied to soil at seven doses: 0.057 (dose recommended by the manufacturer), 1.140, 2.280, 4.560, 9.120, 18.240 and 36.480 mg kg^−1^ soil DM. Uncontaminated soil served as the control treatment. It was found that a mixture of the tested active substances increased the counts of total oligotrophic bacteria and spore-forming oligotrophic bacteria, organotrophic bacteria and actinomycetes, decreased the counts of *Azotobacter* and fungi, and modified the structure of soil microbial communities. The highest values of the colony development (CD) index and the ecophysiological (EP) index were observed in fungi and organotrophic bacteria, respectively. The herbicide applied in the recommended dose stimulated the activity of catalase, urease and acid phosphatase, but it had no effect on the activity of dehydrogenases, alkaline phosphatase, arylsulfatase and β-glucosidase. The highest dose of the analyzed substances (36.480 mg kg^−1^) significantly inhibited the activity of dehydrogenases, acid phosphatase, alkaline phosphatase and arylsulfatase. The values of the biochemical soil fertility indicator (BA_21_) decreased in response to high doses of the herbicide. Urease was most resistant and dehydrogenases were least resistant to soil contamination with a mixture of diflufenican + mesosulfuron-methyl + iodosulfuron-methyl-sodium. The analyzed herbicide had an adverse influence on spring wheat yield, and doses of 18.240 and 36.480 mg kg^−1^ led to eventual death of plants.

## Introduction

Soil, a major component of terrestrial ecosystems, performs numerous important functions in the natural environment. Soil is a source of nutrients for plants and soil-dwelling organisms, it supports biological processes and acts as a buffer to protect groundwater and plants against pollution and prevent translocation of pollutants (Marzaioli et al. 2010). Maintaining and improving soil quality requires a better understanding of soil processes, including biochemistry and microbial activity. Those processes are critical to soil ecosystem functioning since soil microbes play a vital role in organic matter transformation, nutrient cycling and degradation of xenobiotic compounds (Tejada et al. 2011). Soil microbes and enzymes are robust indicators of soil fertility. They promptly respond to environmental changes and therefore adequately reflect biological changes induced by pollution and contamination (Baćmaga et al. 2014b; Cycoń et al. 2012; Panettieri et al. 2013; Tejada 2009).

The use of herbicides is among the factors that contribute to a decrease in soil biological activity. Herbicides are used to increase agricultural production through improving the quality and yield of crops. However, the quantities of the chemicals that do not reach the target organisms are of concern due to their potential impact on the environment (Kucharski et al. 2009). Herbicides that penetrate the soil can affect the population size and activity of soil microbes. Modern herbicides are characterized by high biological activity and selectivity, but their inappropriate and excessive use may have adverse environmental effects (Ayansina and Amusan 2013; Baćmaga et al. 2014a; Bai et al. 2013; Cycoń et al. 2010a; Fenlon et al. 2011; Morgante et al. 2012). The active ingredients of herbicides applied in combination may produce additive or synergistic effects, leading to short-term or long-term changes in the biological equilibrium of soil (Tejada 2009). Soil-dwelling microorganisms are affected not only by active substances but also by their degradation products, which could be more toxic than the parent compounds (Tixier et al. 2002). Some pesticides are slowly degraded in soil, which can lead to their accumulation in concentrations that are harmful for soil-dwelling microorganisms (Muñoz-Leoz et al. 2013). Numerous research studies (Araújo et al. 2003; Cycoń et al. 2013; Muñoz-Leoz et al. 2013) have demonstrated that continued pesticide use can induce changes in soil ecosystems. Pesticides can affect soil microbes, leading to changes in their abundance (Baćmaga et al. 2014b) and biological diversity (Cycoń and Piotrowska-Seget 2009; Cycoń et al. 2010b; Ratcliff et al. 2006) as well as changes in the microbial (Zhang et al. 2010) and enzymatic activity of soil (Baćmaga et al. 2012; Cai et al. 2007; Kucharski and Wyszkowska 2008). Soil microbes are most susceptible to the direct and indirect influence of pesticides in the soil environment, and they are the most robust indicators of environmental stress (Filip 2002). Despite their adverse influence on soil-dwelling microorganisms, pesticides undergo biodegradation, which reduces their toxic environmental effects.

Diflufenican, mesosulfuron-methyl and iodosulfuron-methyl-sodium are active ingredients of the Alister Grande 190 OD herbicide manufactured by Bayer CropScience, which was registered for use in Poland in 2008. Diflufenican (2′,4′-difluoro-2-[α,α,α-trifluoro-*m*-tolyloxy]nicotinanilide) is an active substance that belongs to the group of anilide herbicides. It has a relatively fast rate of degradation and a half-life of 15–30 weeks (Bending et al. 2006). Diflufenican is absorbed by germinating weed seedlings. Its mode of action involves forming a thin layer on the soil surface through which germinating weeds must pass, thus absorbing the chemical (Tejada 2009).

Mesosulfuron-methyl (methyl 2-[[[[(4,6-dimethoxy-2-pyrimidinyl)amino]carbonyl]amino]sulfonyl]-4-[[(methylsulfonyl)amino]methyl]benzoate) and iodosulfuron-methyl-sodium (sodium salt of methyl 4-iodo-2-[[[[(4-methoxy-6-methyl-1,3,5-triazin-2-yl)amino]carbonyl]amino]sulfonyl]benzoate)) are systemic sulfonylurea herbicides that are absorbed by the above-ground parts of weed plants and translocated through the plant. Systemic herbicides affect the activity of the acetolactate synthase (ALS) enzyme, leading to plant deformation and death, growth retardation and chlorosis. They also inhibit the synthesis of branched-chain amino acids and decrease photosynthetic rates (Yuan et al. 2013). Due to their high mobility in the environment, mesosulfuron-methyl and iodosulfuron-methyl-sodium can penetrate into surface and ground waters. Both substances undergo chemical and microbial degradation, and their half-life varies from 38 to 84 days. In the soil environment, sulfonylurea herbicides undergo chemical degradation at pH < 6 and microbiological degradation at pH > 6 (Brigante et al. 2013).

Tejada (2009) analyzed the effect of a mixture of diflufenican and glyphosate on soil microorganisms and determined their degradation rates in two soil types with different granulometric composition. The cited author observed that the microbiological and enzymatic activity of loamy sand and sandy loam protected with glyphosate or diflufenican was higher than in treatments where those herbicides were applied in combination. The above results suggest that a combination of those chemical compounds has a more toxic effect on soil-dwelling microorganisms. The influence of mesosulfuron-methyl and iodosulfuron-methyl-sodium on soil-dwelling organisms has not been studied to date.

The effects of diflufenican, mesosulfuron-methyl and iodosulfuron-methyl-sodium, applied in combination, on soil biological activity remains insufficiently researched. There are no published studies investigating the combined effects of those active substances on soil microorganism and enzymes. The fate and behavior of herbicides in soil are important agri-environmental issues. In view of the above, the objective of this study was to determine the effect of increased doses a mixture of diflufenican + mesosulfuron-methyl + iodosulfuron-methyl-sodium on soil microbial abundance and diversity, activity of soil enzymes and their resistance to the tested product, the biochemical indicator of soil fertility, and spring wheat yield.

## Materials and methods

### Soil

A pot experiment was carried out on samples of soil with the granulometric composition of sandy loam (sand fraction, 72 %; silt fraction, 7 %; clay fraction, 21 %). The analyzed soil had the following parameters: pH_KCl_, 7.0; hydrolytic acidity, 8.00 mmol(+) kg^−1^; total exchangeable bases, 111.00 mmol(+) kg^−1^; organic carbon content, 7.05 g kg^−1^; total nitrogen content, 0.86 g kg^−1^. Samples were collected from the humus horizon, at a depth of 0–20 cm, at the Agricultural Experiment Station in Tomaszkowo owned by the University of Warmia and Mazury in Olsztyn (NE Poland). The soil was classified as Eutric Cambisol based on the World Reference Base of Soil Resources (2008).

### Herbicide

Three active ingredients of the Alister Grande 190 OD herbicide were tested: diflufenican (180.0 g dm^−3^), mesosulfuron-methyl (6.0 g dm^−3^) and iodosulfuron-methyl-sodium (4.5 g dm^−3^). Alister Grande 190 OD is an oil suspension concentrate intended for dilution with water before foliar application. It is used for weed control in winter wheat, winter triticale and rye. The dose recommended by the manufacturer is 0.8 to 1.0 dm^3^ ha^−1^. The active substances are described in the Introduction section.

### Experimental design

A greenhouse experiment was performed in four replications. Polyethylene pots (3.50 dm^3^) were filled with soil passed through a sieve with 2-mm mesh size. Soil was divided into eight equal samples of 3,000 g each. Each sample was treated with a single dose a mixture of diflufenican + mesosulfuron methyl + iodosulfuron-methyl-sodium (0.057 [dose recommended by the manufacturer], 1.140, 2.280, 4.560, 9.120, 18.240, 36.480 mg kg^−1^ soil DM) and mineral fertilizers, soil was thoroughly mixed and placed in pots. The control treatment comprised soil without herbicides. The experiment was carried out in four replications. A total of 32 pots were filled with soil. Increased doses a mixture of diflufenican + mesosulfuron-methyl + iodosulfuron-methyl-sodium were assessed into the soil environment to evaluate the potential threats of their uncontrolled or accidental release into soil. Mineral fertilizers were applied to meet the nutrient requirements of spring wheat, at the following rates (mg kg^−1^ soil, pure active ingredient basis): N, 100 [CO(NH_2_)]_2_; P, 44 [KH_2_PO_4_]; K, 100 [KH_2_PO_4_ + KCl]; Mg, 25 [MgSO_4_⋅7H_2_O]; Cu, 5 [CuSO_4_⋅5H_2_O]; Zn, 5 [ZnCl_2_]; Mn, 5 [MnCl_2_⋅4H_2_O]; Mo, 2.5 [Na_2_MoO_4_⋅2H_2_O]; B, 0.33 [H_3_BO_4_]. The moisture content of soil was brought to 50 % of capillary water capacity using deionized water. Seeds of spring wheat cv. Trape were sown in pots filled with soil (12 plants were left per pot after thinning). Soil moisture content was maintained at a constant level for 60 days of the experiment. On days 30 and 60, soil samples were collected from each pot containing various doses a mixture of diflufenican + mesosulfuron methyl + iodosulfuron-methyl sodium to obtain a bulk sample weighing 400 g for microbiological (five replications per soil sample) and biochemical analyses (three replications per soil sample).

### Soil microorganisms

The counts of total oligotrophic bacteria and spore-forming oligotrophic bacteria were determined in the Onta and Hattori medium (1983) diluted 1:100, organotrophic bacteria — in the Bunt and Rovira medium (Alexander 1973), bacteria of the genus *Azotobacter* — as described by Fenglerowa (1965), actinomycetes — in the Küster and Williams medium with the addition of antibiotics nystatin and actidione (Parkinson et al. 1971), and fungi grown on Martin’s medium (1950) with added Rose Bengal and aureomycin. Plates were kept in a thermostat set to 28 °C throughout the incubation period. The number of colony forming units (CFU) was determined with a colony counter (total oligotrophic bacteria and spore-forming oligotrophic bacteria — after 21 days; actinomycetes and organotrophic bacteria — after 7 days; *Azotobacter* — after 3 days; fungi — after 5 days). Microbial counts were determined on days 30 and 60, in five replications.

The herbicide’s effect on the structure and biodiversity of organotrophic bacteria, actinomycetes and fungi was determined in soil samples. Diluted soil suspensions were incubated in Petri dishes, at 28 °C, in five replications. Colonies were counted over a period of successive 10 days. The results were used to determine the colony development (CD) index: CD = [*N*_1_/1 + *N*_2_/2 + *N*_3_/3 … *N*_10_/10] × 100, where *N*_1_, *N*_2_, *N*_3_, …, *N*_10_ denote the number of colonies that emerged on days 1, 2, 3, …, 10 (Sarathchandra et al. 1997); and the ecophysiological (EP) index: EP = −∑(*p*_*i*_ · log *p*_i_), where *p*_*i*_ is the number of colonies that emerged on a given day divided by the total number of colonies (De Leij et al. 1993).

### Soil enzymes

The activity of dehydrogenase was determined in soil samples by the method of Öhlinger (1996), the activity of acid phosphatase and alkaline phosphatase — by the method of Alef et al. (1998), the activity of catalase, urease, arylsulfatase and β-glucosidase — according to the procedure described by Alef and Nannipieri (1998). The following substrates were used for the determination of enzymatic activity: 2,3,5-triphenyltetrazolium chloride (TTC) for dehydrogenases, 4-nitrophenylphosphate disodium (PNPNa) for phosphatases, urea for urease, *p*-nitrophenyl-β-d-glucopyranoside (PNG) for β-glucosidase, potassium 4-nitrophenyl sulfate (PNS) for arylsulfatase, and hydrogen peroxide for catalase. Enzymatic activity was expressed as follows (kg soil DM h^−1^): μmol triphenyl formazane (TPF) for dehydrogenases, mol O_2_ for catalase, mmol N-NH_4_ for urease, mmol *p*-nitrophenol (PNP) for alkaline phosphatase, acid phosphatase, arylsulfatase and β-glucosidase. Extinction was measured with the Perkin-Elmer Lambda 25 spectrophotometer. The activity of dehydrogenases was measured at a wavelength of 485 nm, the activity of urease, alkaline phosphatase and acid phosphatase — at 410 nm, the activity of arylsulfatase — at 420 nm, and the activity of β-glucosidase — at 400 nm. Catalase activity was identified based on its ability to break down hydrogen peroxide in the presence of potassium permanganate.

The values of soil enzymatic activity were used to calculate the biochemical indicator of soil fertility (BA_21_) based on the equation proposed by Wyszkowska et al. (2013):

$$ {\mathrm{BA}}_{21}=\mathrm{DH}+\mathrm{CA}+\mathrm{U}+\mathrm{PAC}+\mathrm{PAL}+\mathrm{AR}+\mathrm{GL}, $$

where DH is the activity of dehydrogenases, CA is the activity of catalase, U is the activity of urease, PAC is the activity of acid phosphatase, PAL is the activity of alkaline phosphatase, AR is the activity of arylsulfatase, and GL is the activity of β-glucosidase.

Soil resistance (RS) to contamination with the analyzed herbicide was estimated based on the formula proposed by Orwin and Wardle (2004):

$$ \mathrm{RS}=1-\kern0.5em \frac{2\left|{D}_0\right|}{{\mathrm{C}}_0+\left|{D}_0\right|}, $$

where *C*_0_ is the soil resistance under natural conditions over time *t*_0_, and *P*_0_ is the resistance of soil subjected to pressure over time *t*_0_, *D*_0_ = C_0_ − P_0_.

### Spring wheat yield

Twenty-five seeds of spring wheat cv. Trape were sown per pot, and 12 plants were left per pot after thinning. Spring wheat was harvested at the heading stage (BBCH 52; 20 % of inflorescence emerged), and dry matter yield (g pot^−1^) was determined.

### Statistical analysis

Statistical analyses were performed in the Statistica 10.0 application (StatSoft Inc. 2011). Homogeneous subsets of means were identified by Tukey’s range test at a significance level of *p* = 0.05, using ANOVA. Data on soil microbial counts were analyzed by cluster analysis (CA) that involved agglomerative hierarchical clustering. Distances between clusters were measured by Ward’s method, with the Euclidean distance as the distance metric. RS to contamination with a mixture of diflufenican + mesosulfuron-methyl + iodosulfuron-methyl-sodium was determined based on the activity of the analyzed enzymes, by principal component analysis (PCA) using standardized data which meet the assumptions of Pearson's correlation. The results of CA and PCA were processed statistically using multivariate techniques. CA was performed based on microbial counts in five replications for both sampling dates and each herbicide dose. PCA was performed based on average values in three replications for each herbicide dose. Regression equations were derived and coefficients of determination were calculated for the values of the biochemical soil fertility indicator (BA_21_). The percentage of observed variance in soil microbial abundance and enzymatic activity was determined by two-way ANOVA with the use of coefficient *η* ^2^ calculated on the basis of the following formula:

$$ {\eta}^2=\frac{{\mathrm{SS}}_{\mathrm{effect}}}{{\mathrm{SS}}_{\mathrm{total}}}\times 100\%, $$

where *η* ^2^ is the coefficient *η* ^2^, SS_effect_ is the sum of squares corresponding to a given effect and SS_total_ is the sum of squares corresponding to all effects.

## Results

### Soil microorganisms

The abundance of all microbial groups was affected by the dose of diflufenican + mesosulfuron-methyl + iodosulfuron-methyl-sodium as well as the date of the analysis (Table 1). The applied herbicide dose modified microbial abundance by 3.2 % (actinomycetes) to 59.2 % (oligotrophic bacteria), and the date of analysis — by 2.9 % (fungi) to 88.7 % (actinomycetes). Excessive doses of the analyzed herbicide mixture distorted the soil's microbiological balance measured by the abundance of oligotrophic bacteria, spore-forming oligotrophic bacteria, *Azotobacter* spp., organotrophic bacteria, actinomycetes and fungi. In most cases, the dose recommended by the manufacturer (0.057 mg kg^−1^) did not lead to significant changes in the size of the analyzed microbial populations. On both sampling dates, the abundance of oligotrophic bacteria was positively correlated with the dose of the diflufenican + mesosulfuron-methyl + iodosulfuron-methyl-sodium mixture (*r* = 0.85 on day 30 and *r* = 0.77 on day 60). The highest dose of 36.480 mg kg^−1^ had the most stimulating effect on oligotrophic bacteria whose counts increased by 0.36 log on day 30 and by 0.21 log on day 60. The dose recommended by the manufacturer (0.057 mg kg^−1^) decreased the population of oligotrophic bacteria by 0.08 log on day 30. The proliferation of spore-forming oligotrophic bacteria was also determined by herbicide dose and date of analysis. The dose recommended by the manufacturer lowered the counts of the above microbes by 0.18 log on day 30 and by 0.25 log on day 60. The herbicide dose of 36.480 mg kg^−1^ decreased the populations of spore-forming oligotrophic bacteria by 0.13 log on day 30, but it increased their counts by 0.28 log on day 60. *Azotobacter* responded negatively to excessive amounts of the diflufenican + mesosulfuron-methyl + iodosulfuron-methyl-sodium mixture on day 30. The dose of 4.560 mg kg^−1^ lowered *Azotobacter* counts by 0.93 log on day 30, whereas on day 60, the most inhibitory effect was exerted by the dose of 9.120 mg kg^−1^, which decreased the size of the *Azotobacter* population by 0.89 log in comparison with control. The analyzed herbicide mixture stimulated the growth of organotrophic bacteria, and positive correlation coefficients were noted on day 30 and day 60 at *r* = 0.69 and *r* = 0.42, respectively. *Azotobacter* counts were most stimulated by the dose of 36.480 mg kg^−1^, which increased the size of the analyzed population by 0.09 log on day 30 and 0.13 log on day 60. Actinomycetes were also sensitive to the tested herbicide. Higher doses of the analyzed compounds inhibited the proliferation of actinomycetes on day 30. The highest dose of 36.480 mg kg^−1^ lowered actinomycetes counts by 0.08 log on day 30. On day 60, the diflufenican + mesosulfuron-methyl + iodosulfuron-methyl-sodium mixture induced an increase of 0.16 to 0.42 log in the actinomycetes population. The tested herbicide had an adverse influence on fungal growth, and negative correlations were reported between herbicide dose and fungal counts (*r* = −0.57 on day 30 and *r* = −0.30 on day 60). On day 30, the greatest reduction in fungal counts was observed in response to the dose of 0.057 mg kg^−1^, and on day 60 — in response to the dose of 9.120 mg kg^−1^.

**Table 1:** Microbial counts in soil contaminated with a mixture of diflufenican + mesosulfuron-methyl + iodosulfuron-methyl-sodium (D+M+J), log CFU kg^−1^ soil DM

Dose of D+M+J mg kg^−1^	Olig		Oligp		*Az*		Org		Act		Fun	
	Date of analysis (days)											
	30	60	30	60	30	60	30	60	30	60	30	60
0.000	10.11^g^	10.12^fg^	8.65^efg^	8.59^gh^	4.10^bcd^	4.53^a^	10.02^bcde^	10.01^cde^	9.30^fg^	9.80^a^	7.30^abc^	7.22^bcd^
0.057	10.03^h^	10.22^c^	8.83^abc^	8.84^ab^	4.01^cde^	4.44^ab^	10.07^abcd^	10.10^ab^	9.21^h^	10.14^ab^	7.21^bcd^	7.41^a^
1.140	10.13^efg^	10.04^h^	8.74^de^	8.92^a^	3.84^def^	4.29^abc^	10.08^abc^	9.99^de^	9.32^fg^	10.08^bc^	7.32^ab^	7.39^a^
2.280	10.17^de^	10.11^g^	8.64^fg^	8.87^a^	3.43^gh^	4.27^abc^	10.09^abc^	9.98^e^	9.32^fg^	10.06^bcd^	7.32^ab^	7.22^bcd^
4.560	10.32^b^	10.04^h^	8.72^def^	8.73^def^	3.17^h^	3.84^def^	10.09^abc^	9.78^f^	9.34^f^	9.71^e^	7.34^ab^	7.21^bcd^
9.120	10.32^b^	10.16^def^	8.76^cde^	8.74^de^	3.49^fgh^	3.64^efg^	10.09^abc^	10.00^de^	9.30^fg^	9.96^d^	7.30^abc^	7.10^d^
18.240	10.35 ^b^	10.18^cd^	8.71^def^	8.72^def^	3.52^fgh^	4.19^abcd^	10.11^a^	10.02^bcd^	9.22^fg^	9.97^cd^	7.22^bcd^	7.18^cd^
36.480	10.47 ^a^	10.33^b^	8.52^h^	8.87^a^	3.59^fg^	4.40^ab^	10.11^a^	10.14^a^	9.22^fg^	10.22^a^	7.22^bcd^	7.25^bc^
*r*	0.85	0.77	−0.67	0.15	−0.30	0.01	0.69	0.42	−0.57	0.42	−0.57	−0.30

Homogeneous groups are denoted with the same letters within microbial groups for two dates of analysis, in columns

*Olig* oligotrophic bacteria, *Oligp* spore-forming oligotrophic bacteria, *Org* organotrophic bacteria, *Az* bacteria of the genus *Azotobacter*, *Act* actinomycetes, *Fun* fungi, *r* coefficient of correlation

**Fig. 1:**
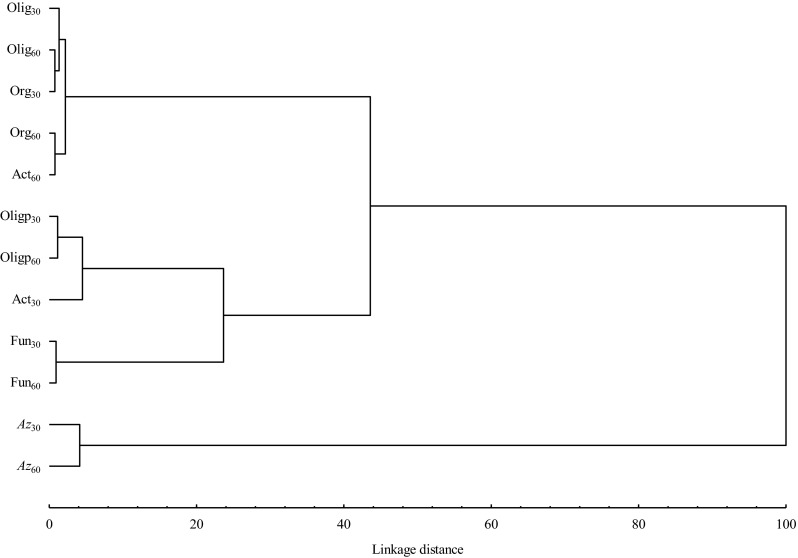
Similar responses of soil-dwelling microorganisms to a mixture of diflufenican + mesosulfuron-methyl + iodosulfuron-methyl-sodium. *Olig* oligotrophic bacteria, *Oligp* spore-forming oligotrophic bacteria, *Az* bacteria of the genus *Azotobacter*, *Org* organotrophic bacteria, *Act* actinomycetes, *Fun* fungi, *30* microbial counts on day 30, *60* microbial counts on day 60

Figure 1 shows a dendrogram grouping soil microorganisms characterized by similar responses to the applied herbicide doses. The dendrogram revealed two clusters of similar microbial groups and a cluster that contained only one element — *Azotobacter*, which was significantly different from the other two. The first group comprised oligotrophic bacteria, organotrophic bacteria identified on days 30 and 60, and actinomycetes identified on day 60. The second group included spore-forming oligotrophic bacteria and fungi identified on days 30 and 60, and actinomycetes identified on day 30. On day 60, organotrophic bacteria and actinomycetes responded similarly to the applied doses of the diflufenican + mesosulfuron-methyl + iodosulfuron-methyl-sodium mixture because the shortest Euclidean distance was noted between those microbial groups. Bacteria of the genus *Azotobacter* were most sensitive to the tested product.

The values of CD and EP indices also changed in response to the application of diflufenican + mesosulfuron-methyl + iodosulfuron-methyl-sodium (Table 2). The highest value of the CD index was observed in fungi (60.60 on average), and the lowest — in actinomycetes (27.25). In organotrophic bacteria and fungi, the value of the CD index was higher on day 30 than on day 60. A reverse correlation was noted in actinomycetes. The CD index was also significantly influenced by herbicide dose. On day 30, organotrophic bacteria were most sensitive to the dose of 0.057 mg kg^−1^ (CD = 30.30), and on day 60 — to the dose of 18.240 mg kg^−1^ (CD = 33.35). In actinomycetes, the lowest value of the CD index was reported on both sampling dates in response to the herbicide dose of 0.057 mg kg^−1^ at 23.60 and 25.40, respectively. The CD index of fungi was lowest in the control treatment at 50.44 on day 30 and 41.00 on day 60.

**Table 2:** Values of the colony development (CD) index and the eco-physiological (EP) index in soil contaminated with a mixture of diflufenican + mesosulfuron-methyl + iodosulfuron-methyl-sodium (D+M+J)

Dose of D+M+J mg kg^−1^	CD						EP					
	Org		Act		Fun		Org		Act		Fun	
	Date of analysis (days)											
	30	60	30	60	30	60	30	60	30	60	30	60
0.000	34.91^bcde^	33.42^cde^	25.17^bc^	26.03^bc^	50.44^defg^	41.00^fg^	0.83^a^	0.82^a^	0.84^abc^	0.87^a^	0.59^a^	0.49^abc^
0.057	30.30^e^	35.72^abcde^	23.60^c^	25.40^bc^	75.57^abc^	40.93^fg^	0.86^a^	0.40^a^	0.76^bcd^	0.87^a^	0.45^abc^	0.36^bc^
1.140	38.24^abc^	35.11^bcde^	24.81^c^	25.93^bc^	81.18^ab^	55.05^defg^	0.86^a^	0.84^a^	0.86^ab^	0.86^ab^	0.37^bc^	0.52^abc^
2.280	35.77^abcde^	33.89^bcde^	25.63^bc^	26.41^bc^	82.68^a^	56.57^cdef^	0.83^a^	0.86^a^	0.85^abc^	0.85^abc^	0.39^abc^	0.48^abc^
4.560	40.82^ab^	42.59^a^	25.25^bc^	38.46^a^	82.09^ab^	62.40^bcde^	0.84^a^	0.82^a^	0.84^abc^	0.60^f^	0.37^bc^	0.47^abc^
9.120	34.82^bcde^	36.15^abcde^	25.27^bc^	30.64^bc^	83.02^a^	41.65^fg^	0.85^a^	0.82^a^	0.77^abcd^	0.81^abcd^	0.32^c^	0.52^abc^
18.240	40.04^abc^	33.35^cde^	25.77^bc^	30.77^b^	66.74^abcd^	43.16^efg^	0.86^a^	0.82^a^	0.77^abcd^	0.80^abcd^	0.50^abc^	0.44^abc^
36.480	37.37^abcd^	36.08^abcde^	25.94^bc^	30.94^b^	57.41^cdef^	49.73^defg^	0.83^a^	0.78^b^	0.74^abc^	0.66^ef^	0.54^ab^	0.47^abc^
*r*	0.33	−0,02	0.61	0.33	−0.42	−0.07	−0.29	−0.82	−0.69	−0.55	0.35	−0.02

Homogeneous groups are denoted with the same letters within microbial groups for two dates of analysis, in columns

*Org* organotrophic bacteria, *Act* actinomycetes, *Fun* fungi, *r* coefficient of correlation

The values of the EP index indicate that the diflufenican + mesosulfuron-methyl + iodosulfuron-methyl-sodium mixture had a significant influence on the biological diversity of organotrophic bacteria, actinomycetes and fungi. On average, the highest EP index was reported for organotrophic bacteria (EP = 0.84), and the lowest — for fungi (EP = 45). Organotrophic bacteria and actinomycetes were characterized by a higher EP index on day 30 than on day 60, whereas a reverse correlation was noted in fungi. The analyzed herbicide induced minor changes in the biological diversity of organotrophic bacteria, and the lowest value of the EP index at 0.78 was observed on day 60 in treatments subjected to the dose of 36.480 mg kg^−1^. The tested compounds had a negative influence on the biological diversity of actinomycetes. On day 30, the dose of 36.480 mg kg^−1^ lowered the value of the EP index by 11.9 %, and on day 60, the value of the EP index decreased by 31.0 % in response to the dose of 4.560 mg kg^−1^. The tested herbicide had a varied influence on the EP index of fungi. On day 30, the greatest reduction in the value of the EP index was reported in treatments subjected to the dose of 9.120 mg kg^−1^ (45.8 %), and on day 60 — in treatments subjected to the dose of 0.057 mg kg^−1^ (26.5 %).

### Soil enzymes

The mixture of diflufenican + mesosulfuron-methyl + iodosulfuron-methyl-sodium had varied effects on soil enzymatic activity (Table 3). The activity of soil enzymes was significantly influenced by the herbicide dose (the percentage of variance ranged from 5.2 % to 51.5 %) and date of analysis (the percentage of variance ranged from 3.0 % to 85.5 %). On day 30, the dose recommended by the manufacturer induced an increase in the activity dehydrogenases (by 11.8 %), catalase (by 50 %), urease (by 30.1 %), acid phosphatase (by 10.3 %) and arylsulfatase (by 11.1 %). On day 60, the above dose exerted a stimulating effect on acid phosphatase and alkaline phosphatase whose activity increased by 14.1 % and 14.5 %, respectively, relative to control. Higher doses of the herbicide adversely affected the activity of dehydrogenases, which was reflected by negative values of the correlation coefficient between herbicide dose and dehydrogenase activity (*r* = −0.37 on day 30 and *r* = −0.73 on day 60). Dehydrogenase activity was most inhibited by the dose of 9.120 mg kg^−1^ (decrease of 10.1 %) on day 30 and by the dose of 36.480 mg kg^−1^ (decrease of 62.6 %) on day 60. The diflufenican + mesosulfuron-methyl + iodosulfuron-methyl-sodium mixture had a varied effect on catalase activity. On day 30, catalase activity in treatments subjected to doses of 0.057 to 36.480 mg kg^−1^ increased by 21.4–50.0 %, but on day 60 catalase activity decreased in response to all doses, and the greatest reduction of 87.5 % was observed in response to the dose of 4.560 mg kg^−1^. The analyzed herbicide also influenced urease activity. On day 30, the diflufenican + mesosulfuron-methyl + iodosulfuron-methyl-sodium mixture stimulated urease activity in comparison with control, excluding in treatments subjected to the dose of 4.560 mg kg^−1^, where urease activity decreased by only 3.2 %. On day 60, the greatest decrease in urease activity of 13.2 % was induced by the highest herbicide dose of 36.480 mg kg^−1^. Acid phosphatase also proved to be sensitive to the combination of diflufenican + mesosulfuron-methyl + iodosulfuron-methyl-sodium. Acid phosphatase activity was negatively correlated with herbicide dose on both dates of analysis (*r* = −0.92 on day 30 and *r* = −0.13 on day 60). Alkaline phosphatase responded negatively to soil contamination with the diflufenican + mesosulfuron-methyl + iodosulfuron-methyl-sodium mixture. On both sampling dates, the herbicide dose of 36.480 mg kg^−1^ exerted the most inhibitory effect on the above enzyme whose activity decreased by 12.4 % and 19.3 %, respectively. The tested herbicide also modified arylsulfatase activity, which ranged from 0.25 to 0.30 mmol PNP kg^−1^ DM h^−1^ on day 30, and from 0.34 to 0.41 mmol PNP kg^−1^ DM h^−1^ on day 60. β-Glucosidase was also sensitive to the combination of diflufenican + mesosulfuron-methyl + iodosulfuron-methyl-sodium. On day 30, β-glucosidase activity across experimental treatments was fairly similar in the range of 0.29 to 0.31 mmol PNP kg^−1^ DM h^−1^. On day 60, herbicide doses of 1.140 and 2.280 mg kg^−1^ lowered β-glucosidase activity by 13.8 %.

**Table 3:** Enzymatic activity in soil contaminated with a mixture of diflufenican + mesosulfuron-methyl + iodosulfuron-methyl-sodium (D+M+J), 1 kg DM h^−1^

Dose of D+M+J mg kg^−1^	DH		CA		U		PAC		PAL		AR		GL	
	μmol TPF		mol O_2_		mmol N-NH_4_		mmol PNP							
	Date of analysis (days)													
	30	60	30	60	30	60	30	60	30	60	30	60	30	60
0.000	12.19^cde^	14.46^a^	0.14^efg^	0.24^a^	0.93^cd^	0.68^ef^	2.90^ef^	4.41^bc^	5.81^a^	4.14^e^	0.27^de^	0.41^a^	0.31^a^	0.29^ab^
0.057	13.63^ab^	13.96^ab^	0.21^abc^	0.23^ab^	1.21^a^	0.64^efg^	3.20^e^	5.03^a^	5.14c	4.70^d^	0.30^cde^	0.41^a^	0.30^a^	0.27^abc^
1.140	12.06^cdef^	13.12^bc^	0.19^cde^	0.19^cde^	0.98^bc^	0.64^efg^	3.03ef	4.73^ab^	5.23^bc^	4.21^e^	0.29^cde^	0.38^ab^	0.29^ab^	0.25^d^
2.280	12.43^cd^	8.56^g^	0.21^abc^	0.04^h^	0.95^bcd^	0.65^efg^	2.91^ef^	3.24^e^	5.58^ab^	4.12^e^	0.29^cde^	0.40^ab^	0.30^a^	0.25^d^
4.560	11.56^def^	7.80^g^	0.17^def^	0.03^h^	0.90^d^	0.71^e^	2.85^ef^	3.18^e^	5.76^a^	3.82^ef^	0.30^cde^	0.39^ab^	0.30^a^	0.27^abc^
9.120	10.96^f^	6.37^h^	0.19^cde^	0.14^efg^	1.00^b^	0.70^ef^	2.74^fg^	3.71^d^	5.51^abc^	3.43^fg^	0.28^de^	0.40^ab^	0.29^ab^	0.29^ab^
18.240	11.03^ef^	5.64^h^	0.18^de^	0.24^a^	1.00^b^	0.60^g^	2.69^fg^	3.94^d^	5.18^bc^	3.45^fg^	0.27^de^	0.34^bcd^	0.30^a^	0.29^ab^
36.480	11.97^cdef^	5.41^h^	0.20^bcd^	0.18^de^	1.00^b^	0.59^g^	2.33^g^	4.11^cd^	5.09^cd^	3.34^g^	0.25^e^	0.36^abc^	0.30^a^	0.29^ab^
*r*	−0.37	−0.73	0.22	0.13	−0.05	−0.66	−0.92	−0.13	−0.52	−0.77	−0.86	−0.74	0.04	0.43

Homogeneous groups are denoted with the same letters within soil enzymes for two dates of analysis, in columns

*DH* dehydrogenases, *CA* catalase, *U* urease, *PAC* acid phosphatase, *PAL* alkaline phosphatase, *AR* arylsulfatase, *GL* β-glucosidase, *r* coefficient of correlation

**Fig. 2:**
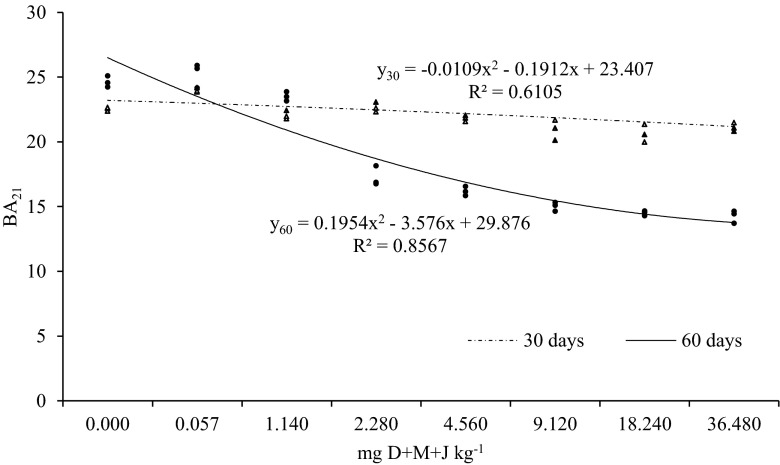
Biochemical indicator of soil fertility (BA21) in soil contaminated with a mixture of diflufenican + mesosulfuron-methyl + iodosulfuron-methyl-sodium (D+M+J)

The values of the biochemical soil fertility indicator (BA_21_) decreased in response to diflufenican + mesosulfuron-methyl + iodosulfuron-methyl-sodium applied in high doses (Fig. 2). On day 30, the greatest decrease (8.4 %) in the value of BA_21_ was noted in response to the herbicide dose of 18.240 mg kg^−1^, and on day 60 — in response to the dose of 36.480 mg kg^−1^ (decrease of 42.1 %). The dose recommended by the manufacturer did not induce significant changes — the value of BA_21_ increased by 6.4 % on day 30 and by only 4.1 % on day 60 relative to control. Regardless of the applied herbicide dose, the value of the biochemical soil fertility indicator was 1.2-fold higher on day 30 than on day 60.

The RS values shown in Table 4 indicate that soil enzymes varied in their sensitivity to contamination with a mixture of diflufenican + mesosulfuron-methyl + iodosulfuron-methyl-sodium. The highest average RS value was noted for urease (0.929), and the lowest — for dehydrogenases (0.635). Regardless of the applied herbicide dose, enzyme resistance to contamination was generally higher on day 30 than on day 60, excluding catalase and arylsulfatase whose resistance on day 60 was higher by 26.2 % and 5.9 %, respectively, than on day 30. Dehydrogenases’ resistance to the diflufenican + mesosulfuron-methyl + iodosulfuron-methyl-sodium mixture was determined by the applied dose and date of analysis. On day 30, RS values ranged from 0.79 (0.057 mg kg^−1^ dose) to 0.96 (36.480 mg kg^−1^ dose). On day 60, the value of the RS index decreased with a drop in herbicide dose. The highest dose of 36.480 mg kg^−1^ lowered RS values by 74.7 %. The tested herbicide had a varied effect on catalase resistance. On day 30, the lowest levels of catalase resistance were reported in response to the dose of 2.280 mg kg^−1^ (RS = 0.30), and on day 60 — in response to the dose of 4.560 mg kg^−1^ (RS = 0.08). Urease resistance to soil contamination with the tested herbicide varied over time. On day 30, the RS values of urease were higher in treatments subjected to high doses of the diflufenican + mesosulfuron-methyl + iodosulfuron-methyl-sodium mixture, and the greatest reduction in resistance (by 79.2 %) was induced by the dose of 2.280 mg kg^−1^. On day 30, the lowest level of urease resistance was noted in treatments subjected to the herbicide dose of 36.480 mg kg^−1^ (RS = 0.78). Acid phosphatase also responded to soil contamination with the combination of diflufenican + mesosulfuron-methyl + iodosulfuron-methyl-sodium. On day 30, herbicide doses of 0.057 to 18.240 mg kg^−1^ led to a 4.9–20.7 % increase in the resistance of acid phosphatase. The highest dose of 36.480 mg kg^−1^ lowered the RS index of acid phosphatase by 18.3 % on day 30, but it increased RS value by 17.3 % on day 60. On day 60, the highest, 1.3-fold reduction in the RS value of acid phosphatase was reported in response to the dose of 4.560 mg kg^−1^. The values of the RS index of alkaline phosphatase were determined in the range of 0.68 to 0.97. On both dates of analysis, RS values were negatively correlated with herbicide dose. Alkaline phosphatase was characterized by the lowest resistance in treatments subjected to the dose of 36.480 mg kg^−1^ (RS = 0.78 on day 30 and RS = 0.68 on day 60). The resistance of arylsulfatase to soil contamination was determined by both herbicide dose and sampling date. On day 60, the RS value of the above enzyme was negatively correlated with herbicide dose (*r* = −0.77). The herbicide dose of 36.480 mg kg^−1^ lowered the RS index by 12.1 % on day 60. The resistance of β-glucosidase to soil contamination with the diflufenican + mesosulfuron-methyl + iodosulfuron-methyl-sodium mixture was similar across the analyzed treatments on day 30 (0.92 to 0.97).

**Table 4:** Resistance of soil enzymes to contamination with a mixture of diflufenican + mesosulfuron-methyl + iodosulfuron-methyl-sodium (D+M+J)

Dose of D+M+J mg kg^−1^	DH		CA		U		PAC		PAL		AR		GL	
	Date of analysis (days)													
	30	60	30	60	30	60	30	60	30	60	30	60	30	60
0.057	0.79^b^	0.91^ab^	0.32^def^	0.92^a^	0.53^c^	0.91^ab^	0.82^abc^	0.75^bcd^	0.80^cdef^	0.76^def^	0.79^a^	0.58^b^	0.96^a^	0.88^ab^
1.140	0.95^a^	0.83^ab^	0.41^bcde^	0.64^b^	0.88^ab^	0.88^ab^	0.92^ab^	0.86^abc^	0.82^abcdef^	0.94^ab^	0.89^a^	0.58 ^b^	0.92^a^	0.75^bc^
2.280	0.95^a^	0.42^c^	0.30^efg^	0.14^fg^	0.95^a^	0.93^ab^	0.99^a^	0.58^d^	0.93^abc^	0.95^ab^	0.88^a^	0.58^b^	0.97^a^	0.72^c^
4.560	0.90^ab^	0.37^cd^	0.62^bc^	0.08^g^	0.94^a^	0.92^ab^	0.90^ab^	0.56^d^	0.97^a^	0.85^abcde^	0.82^a^	0.52^b^	0.96 ^a^	0.85^ab^
9.120	0.82^ab^	0.28^cd^	0.44^bcde^	0.44^bcde^	0.86^ab^	0.95^a^	0.88^abc^	0.73^bcd^	0.90^abcd^	0.71^ef^	0.89^a^	0.56^b^	0.93 ^a^	0.94^a^
18.240	0.83^ab^	0.24^d^	0.50^bcde^	0.95^a^	0.86^ab^	0.80^ab^	0.86^abc^	0.81^abc^	0.81^bcdef^	0.71^ef^	0.88^a^	0.55^b^	0.94^a^	0.95^a^
36.480	0.96^a^	0.23^d^	0.37^cdef^	0.58^bcd^	0.86^ab^	0.78^ab^	0.67^cd^	0.88^abc^	0.78^cdef^	0.68^f^	0.84^a^	0.51^b^	0.97^a^	0.95^a^
*r*	0.22	−0.67	0.02	0.19	0.16	−0.82	−0.82	0.50	−0.45	−0.70	0.01	−0.77	0.27	0.66

Homogeneous groups are denoted with the same letters within soil enzymes for two dates of analysis, in columns

*DH* dehydrogenases, *CA* catalase, *U* urease, *PAC* acid phosphatase, *PAL* alkaline phosphatase, *AR* arylsulfatase, *GL* β-glucosidase, *r* coefficient of correlation

**Fig. 3:**
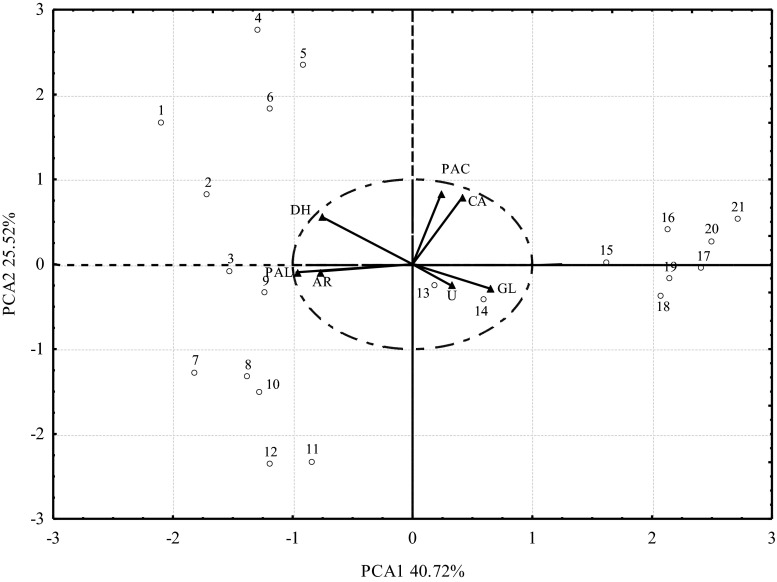
Resistance of soil enzymes to contamination with a mixture of diflufenican + mesosulfuron-methyl + iodosulfuron-methyl-sodium (D+M+J), determined by PCA. Vectors represent the analyzed variables: *DH* dehydrogenases, *CA* catalase, *U* urease, *PAC* acid phosphatase, *PAL* alkaline phosphatase, *AR* arylsulfatase, *GL* β-glucosidase; points represent soil samples contaminated with different doses of D+M+J mg kg^−1^: 0.057 (1, 2, 3), 1.140 (4, 5, 6), 2.280 (7, 8, 9), 4.560 (10, 11, 12), 9.120 (13, 14, 15), 18.240 (16, 17, 18), 36.480 (19, 20, 21)

Figure 3 illustrates the distribution of variance between the first two principal components. The horizontal axis and the vertical axis explain 40.7 % and 25.5 % of total variance, thus explaining 66.2 % of variance of the original variables. The vectors corresponding to the original variables for the resistance of dehydrogenases, alkaline phosphatase, acid phosphatase and catalase are positioned closest to the boundaries of the unit circle, and therefore they are represented by the first two principal components that define the coordinate system. Two homogeneous groups were identified for the first principal component. The first group comprised urease and β-glucosidase, and the second group consisted of dehydrogenases, alkaline phosphatase and arylsulfatase. The resistance of the above enzymes was positively correlated with the first principal component. The homogeneous group for the second principal component comprised catalase and acid phosphatase whose resistance was positively correlated with the analyzed variable. The resistance of alkaline phosphatase, arylsulfatase and dehydrogenases was negatively correlated with doses of diflufenican + mesosulfuron-methyl + iodosulfuron-methyl-sodium. The results of the analysis show that soil contamination with a mixture of diflufenican + mesosulfuron-methyl + iodosulfuron-methyl-sodium affected the resistance of soil enzymes, as indicated by the vectors that lie along the axes of the coordinate system.

### Spring wheat yield

**Fig. 4:**
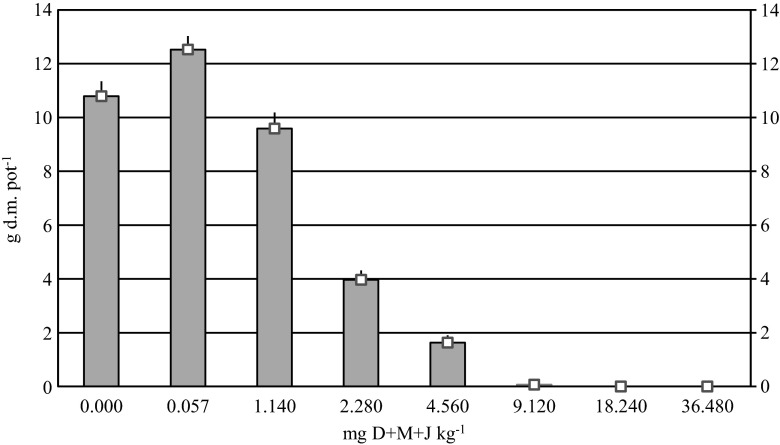
Yield of spring wheat grown in soil contaminated with a mixture of diflufenican + mesosulfuron-methyl + iodosulfuron-methyl-sodium (D+M+J), g pot^−1^

Increased doses of diflufenican + mesosulfuron-methyl + iodosulfuron-methyl-sodium inhibited the growth of spring wheat and decreased its yield (Fig. 4). The tested active substances applied at the recommended dose contributed to a 16.1 % increase in spring wheat yield, compared with the control treatment. Herbicide doses of 1.140–9.120 mg kg^−1^ decreased wheat yield by 11.1–99.3 %. The doses of 18.240 and 36.480 mg kg^−1^ exerted the strongest inhibitory effect and completely inhibited wheat growth.

## Discussion

### Soil microorganisms

Herbicides may affect the counts and biodiversity of soil microorganisms that participate in processes responsible for soil fertility. Soil quality is inextricably linked to microbial transformations; hence, there is a need for a better understanding and a thorough analysis of those processes (Bello et al. 2008; Saha et al. 2012). The results of our study indicate that a mixture of diflufenican + mesosulfuron-methyl + iodosulfuron-methyl-sodium had varied effects on the analyzed soil microorganisms. Soil-dwelling microorganisms respond differently to pesticides and their doses, but differences are also noted across various species of microbial populations colonizing the soil environment (Cycoń et al. 2013). The average values noted in this study indicate that the diflufenican + mesosulfuron-methyl + iodosulfuron-methyl-sodium mixture had a generally stimulating effect on oligotrophic bacteria, spore-forming oligotrophic bacteria, organotrophic bacteria and actinomycetes. The above microorganisms probably rely on herbicides as sources of nutrients and energy (Cycoń et al. 2013). An increase in the counts of bacteria and actinomycetes was also noted by Martinez et al. (2008) in response to sulfentrazone treatment in a dose of 0.7 μg g^−1^ soil. In a study by Araújo et al. (2003), glyphosate doses of 2.16 mg kg^−1^ increased actinomycetes counts, but decreased the size of bacterial populations. Cycoń and Piotrowska-Seget (2009) reported an increase in the size of heterotrophic bacterial populations in loamy sand contaminated with diuron herbicide at doses of 1.5, 7.5 and 150 mg kg^−1^ soil. Ratcliff et al. (2006) did not observe any changes in microbial abundance in soil treated with a glyphosate dose of 50 mg kg^−1^, but microbial counts increased in response to a 100-fold increase in herbicide dose. In a study by Kucharski and Wyszkowska (2008), the Apyros 75 WG herbicide at doses of 8.9, 89.0 and 890.0 μg kg^−1^ containing sulfosulfuron inhibited the proliferation of bacteria and actinomycetes, and bacteria were most sensitive to the tested compound. The inhibitory effects of herbicides on soil-dwelling microorganisms were also confirmed by Sebiomo et al. (2011) in whose experiment, soil was contaminated with atrazine, primextra, paraquat and glyphosate. The average results reported in this study indicate that the tested herbicide inhibited the proliferation of *Azotobacter* and fungi. *Azotobacter* strains were most sensitive to the treatment, which corroborates the findings of Milošević and Govedarica (2002) who demonstrated that bacteria of the genus *Azotobacter* are effective indicators of pesticide soil contamination. The observed decrease in the abundance of the analyzed microorganisms in soil contaminated with the diflufenican + mesosulfuron-methyl + iodosulfuron-methyl-sodium mixture could be attributed to the production of chemical metabolites that exert toxic effects on microbial cells. In the work of Kucharski and Wyszkowska (2008), fungal proliferation was inhibited by the Apyros 75 WG herbicide. A stimulatory effect of herbicides on fungal counts was observed by Crouzet et al. (2010) in a study of mesotrione applied to soil at 0.45 to 45 mg kg^−1^, and by Zabaloy et al. (2010) in a study of 2,4-dichlorophenoxyacetate applied to soil at 1 to 10 mg kg^−1^. Martinez et al. (2008) found that fungi were not affected by sulfentrazone.

Pesticides influence the abundance of soil-dwelling microorganisms and induce changes in the structure of microbial communities (Baćmaga et al. 2014a; Cycoń and Piotrowska-Seget 2009; Cycoń et al. 2012; Ratcliff et al. 2006). In this study, the biodiversity of microbial populations was evaluated with the use of the EP diversity index and the CD index. According to Cycoń and Piotrowska-Seget (2009) and Cycoń et al. (2012), those indices provide valuable information about the influence of pesticides on the biological diversity of soil and the resulting proportions of r-strategists and K-strategists.

The highest average value of the EP index was noted in organotrophic bacteria, and the lowest value was reported in fungi. The results of this study imply that pesticide-induced changes in the soil environment can significantly influence the proliferation of both slow-growing and fast-growing microbes. In a study by Cycoń and Piotrowska-Seget (2009), diuron herbicide decreased the biological diversity of soil-dwelling microorganisms in treatments subjected to doses of 1.5, 7.5 and 150 mg kg^−1^. Soil samples containing diuron were characterized by a predominance of K-strategists, excluding the treatment subjected to a dose of 7.5 mg kg^−1^ where a higher proportion of r-strategists was reported. The application of the linuron herbicide at doses of 4, 20 and 400 mg kg^−1^ disrupted the biological diversity of microbial communities in loamy sand and sandy loam (Cycoń et al. 2010b). The highest dose of 400 mg kg^−1^ increased the number of K-strategists in loamy sand and contributed to a greater abundance of r-strategists in sandy loam. Ros et al. (2006) also observed lower levels of microbial diversity after the application of atrazine at doses of 100 and 1,000 mg kg^−1^. In our study, a mixture of diflufenican + mesosulfuron-methyl + iodosulfuron-methyl-sodium increased the value of the CD index relative to the control sample. The highest average value of the CD index was noted in fungi, and the lowest in actinomycetes. The observed increase in the value of the CD index points to a predominance of r-strategists over K-strategists in soil. The tested product also contributed to changes in microbial biodiversity determined based on the value of the EP index. Baćmaga et al. (2014a) also observed changes in the structure and biodiversity of microbial communities after the application of metazachlor doses of 0.333 to 213.312 mg kg^−1^ soil DM.

### Soil enzymes

Enzymes participate in the biodegradation of natural and anthropogenic organic compounds in soil, and are often used as indicators of changes that occur in the soil environment in response to crop protection chemicals, including herbicides (Baćmaga et al. 2012; Gianfreda et al. 2005; Singh and Kumar 2008; Wang et al. 2009). In the current study, soil enzymes showed different sensitivity to a mixture of diflufenican + mesosulfuron-methyl + iodosulfuron-methyl-sodium. The applied herbicide had varied effects on soil enzymatic activity, compared with uncontaminated samples. According to Gomez et al. (2009), dehydrogenase activity is the most robust indicator of the physiological status of soil microbes because dehydrogenases are present in all living cells. In our study, dehydrogenases were most sensitive to soil contamination with the diflufenican + mesosulfuron-methyl + iodosulfuron-methyl-sodium mixture. The tested herbicide had the most inhibitory effect on dehydrogenases at the dose of 36.480 mg kg^−1^. Cycoń et al. (2013) demonstrated that dehydrogenases were strongly inhibited by napropamide doses of 2.25 and 22.5 mg kg^−1^. The inhibitory effect produced by the 2.25 mg kg^−1^ dose was transient, and on incubation day 28, dehydrogenase activity was similar to that of control soil. Doses 10-fold higher than those recommended by the manufacturer significantly inhibited dehydrogenase activity throughout the experiment. An inhibitory effect of the Apyros 75 WG herbicide on dehydrogenases was also reported by Kucharski and Wyszkowska (2008). Baćmaga et al. (2012) did not observe significant changes in dehydrogenase activity in soil contaminated with the Aurora 40 WG herbicide. Dehydrogenases, similarly as catalase, are intracellular enzymes whose activity is strongly correlated with microbial activity (Bello et al. 2013). However, in our study catalase and dehydrogenases differed in their responses to soil contamination with a mixture of diflufenican + mesosulfuron-methyl + iodosulfuron-methyl-sodium. Catalase activity was significantly reduced on day 60 in response to herbicide doses of 2.280 to 9.120 mg kg^−1^. Yao et al. (2006) did not report significant changes in catalase activity after the application of acetamiprid at doses of 0.5, 5 and 50 mg kg^−1^. The different response of catalase and dehydrogenases to soil contamination with diflufenican + mesosulfuron-methyl + iodosulfuron-methyl-sodium could result from abiotic reactions leading to hydrogen peroxide decomposition, which could mask the response of catalase to the analyzed active substances. Extracellular catalase, also found in soil, is more stable than intracellular catalase, due to its sorption on the surface of clay minerals and associations with organic soil colloids (Bello et al. 2013; Calamai et al. 2000).

In this study, the activity of hydrolase enzymes was determined by the applied herbicide dose and date of analysis. A decrease in phosphatase activity was also noted by Yao et al. (2006) in soil contaminated with acetamipirid, by Wyszkowska and Kucharski (2004) in a study of Triflurotox 250 EC, and by Wyszkowska (2002) in an experiment with Treflan 460 EC. Baćmaga et al. (2012) observed a positive effect of the Aurora 40 WG herbicide containing carfentrazone-ethyl on the activity of the above enzymes. Arylsulfatase responded negatively to soil contamination with diflufenican + mesosulfuron-methyl + iodosulfuron-methyl-sodium. The lowest level of arylsulfatase activity (0.25 mmol PNP kg^−1^ DM h^−1^) was reported on day 30 in response to the highest herbicide dose of 36.480 mg kg^−1^. Similar results were reported by Sofo et al. (2012) who studied the effects of cinosulfuron, prosulfuron, thifensulfuron-methyl and triasulfuron, and by Sukul (2006) in an experiment with metalaxyl.

Pesticides can also influence physiological processes in microorganisms, such as cell lysis and changes in the cell membrane, thus modifying the activity of soil enzymes (Gonod et al. 2006; Hussain et al. 2009; Romero et al. 2010). The stimulatory and inhibitory effects of diflufenican + mesosulfuron-methyl + iodosulfuron-methyl-sodium, was observed in this study. In the work of Tejada (2009), the microbiological activity of loamy sand and sandy loam was inhibited by a combination of glyphosate and diflufenican, whereas no such effect was observed in treatments where the tested herbicides were applied separately. Selected soil enzymes may have a varied response to pesticides (Acosta-Martinez et al. 2008). In the present study, the tested herbicide had a neutral effect on the activity of urease and β-glucosidase. In treatments subjected to the highest herbicide dose, urease activity increased by 7.5 % on day 30 and decreased by 13.2 % on day 60. On day 30, the activity levels of β-glucosidase were fairly similar across the analyzed treatments, but on day 60, they decreased by 13.8 % in treatments exposed to herbicide doses of 1.140 and 2.280 mg kg^−1^. Saha et al. (2012) observed higher levels of β-glucosidase activity in soil samples treated with alachlor, butachlor and pretilachlor. Contrary results were reported by Perucci et al. (1999). The cited authors noted an inhibitory effect of rimsulfuron on β-glucosidase activity, in particular when the herbicide was applied in a dose 100-fold higher than recommended by the manufacturer. Sukul (2006) demonstrated that metalaxyl inhibited urease activity. In a study by Baćmaga et al. (2012) who investigated the effect of the Aurora 40 WG herbicide in two types of soil, urease activity levels decreased in sandy loam and increased slightly in loamy sand. The sensitivity of urease to herbicide pollution was also reported by Kucharski and Wyszkowska (2008) who analyzed the effects of Apyros 75 WG applied to soil. Our findings suggest that soil enzymes varied in their responses to soil contamination with a mixture of diflufenican + mesosulfuron-methyl + iodosulfuron-methyl-sodium.

A soil ecosystem is considered stable if it is able to resist destabilizing stressors or quickly recover its healthy state (Griffiths and Philippot 2013; Orwin and Wardle 2004). Soil stability is usually determined based on resistance and resilience. In the current study, RS values were calculated to estimate the resistance of sandy loam to stress caused by contamination with diflufenican + mesosulfuron-methyl + iodosulfuron-methyl-sodium. Urease was found to be most resistant to the tested herbicide, and dehydrogenases were least resistant to soil contamination In a study investigating the effect of the 2,4-D herbicide applied to the soil at 36 mg kg^−1^, Bécaert et al. (2006) observed that β-glucosidase and arylsulfatase were most sensitive to the above product. According to Griffiths and Philippot (2013), a stressed soil ecosystem becomes more stable after long-term exposure to environmental stressors, which contributes to the development of defense mechanisms responsible for maintaining biological stability.

### Spring wheat yield

Crop protection chemicals are widely used to maintain the adequate quality and health of plants (Martins et al. 2007). However, inappropriate use of those products may suppress plant growth and development, thus decreasing crop yields (Andrea et al. 2003 ). A mixture of diflufenican + mesosulfuron-methyl + iodosulfuron-methyl-sodium had an adverse effect on the grain yield of spring wheat, which was proportional to the applied dose. The herbicide, penetrating into plant tissues in high amounts, could reduce nutrient uptake and photosynthetic rates, leading to wheat growth inhibition. The chemical structure of the analyzed active substances could also affect wheat yield, which corroborates the findings of Kucharski and Wyszkowska (2008) who analyzed the Apyros 75 WG herbicide. Diflufenican + mesosulfuron-methyl + iodosulfuron-methyl-sodium, applied in combination, had an adverse influence on spring wheat yield, and doses of 18.240 and 36.480 mg kg^−1^ led to the eventual death of plants.

## Conclusions

A mixture of three active substances — diflufenican, mesosulfuron-methyl and iodosulfuron-methyl-sodium — exerted varied effects on the microbiological and enzymatic properties of soil. When applied at increased doses, the above herbicide increased the counts of oligotrophic bacteria, spore-forming oligotrophic bacteria, organotrophic bacteria and actinomycetes, but decreased the populations of bacteria of the genus *Azotobacter* and fungi. Diflufenican + mesosulfuron-methyl + iodosulfuron-methyl-sodium modified the structure of soil microbial communities, leading to changes in CP and EP indices. The highest values of CD and EP were observed in fungi and organotrophic bacteria, respectively. At contaminating doses, the tested product suppressed the activity of dehydrogenases, acid phosphatase, alkaline phosphatase and arylsulfatase, and it had the most inhibitory effect on dehydrogenases. The values of the biochemical soil fertility indicator (BA_21_) decreased considerably in response to diflufenican + mesosulfuron-methyl + iodosulfuron-methyl-sodium applied in high doses. Urease was most resistant and dehydrogenases were least resistant to soil contamination with a mixture of diflufenican + mesosulfuron-methyl + iodosulfuron-methyl-sodium. In general, increased doses of diflufenican + mesosulfuron-methyl + iodosulfuron-methyl-sodium had a more inhibitory effect on the biological activity of soil on day 60 than on day 30.
